# Reconstruction of a Genome-scale Metabolic Network of *Komagataeibacter nataicola* RZS01 for Cellulose Production

**DOI:** 10.1038/s41598-017-06918-1

**Published:** 2017-08-11

**Authors:** Heng Zhang, Chao Ye, Nan Xu, Chuntao Chen, Xiao Chen, Fanshu Yuan, Yunhua Xu, Jiazhi Yang, Dongping Sun

**Affiliations:** 1 Chemicobiology and Functional Materials Institute, Nanjing University of Science and Technology, Nanjing, 210094 China; 2 School of Chemical Engineering, Nanjing University of Science and Technology, Nanjing, 210094 China; 3 State Key Laboratory of Food Science and Technology, Jiangnan University, Wuxi, 214122 China; 4 Department of Life Sciences, Lianyungang Normal College, Lianyungang, 222000 China

## Abstract

Bacterial cellulose (BC) is widely used in industries owing to its high purity and strength. Although *Komagataeibacter nataicola* is a representative species for BC production, its intracellular metabolism leading to BC secretion is unclear. In the present study, a genome-scale metabolic network of cellulose-producing *K. nataicola* strain RZS01 was reconstructed to understand its metabolic behavior. This model *i*HZ771 comprised 771 genes, 2035 metabolites, and 2014 reactions. Constraint-based analysis was used to characterize and evaluate the critical intracellular pathways. The analysis revealed that a total of 71 and 30 genes are necessary for cellular growth in a minimal medium and complex medium, respectively. Glycerol was identified as the optimal carbon source for the highest BC production. The minimization of metabolic adjustment algorithm identified 8 genes as potential targets for over-production of BC. Overall, model *i*HZ771 proved to be a useful platform for understanding the physiology and BC production of *K. nataicola*.

## Introduction

Acetic acid bacteria (AAB) are a group of aerobic, gram-negative bacteria belonging to class α-Proteobacteria^1^. They have been isolated from a variety of natural sources, including fruits, fermented foods, plant organs, and soil^2–4^. *Komagataeibacter* and *Acetobacter* are the main genera of AAB, and have been widely used in several industrial processes, such as acetic acid production and cocoa bean fermentation^4, 5^. Besides, these bacteria have been employed in chemical industries for the production of ascorbic acid and bacterial cellulose (BC)^6, 7^.

*Komagataeibacter nataicola* (formerly known as *Gluconacetobacter xylinus*) has been reported as a high-yield cellulose-producing strain^8^. The strain possesses a number of remarkable physiological properties, such as the ability to oxidize a wide range of substrates and to tolerate high concentrations of acetic acid and ethanol^9^. The ability to produce BC is one of the most interesting features of this strain^5^. Although, BC has a primary chemical composition similar to that of the cellulose derived from plants and algae, its excellent structural and mechanical characteristics make it a valuable resource for industrial applications^10–12^. Nonetheless, low fermentation yields and high cost of production are the main bottlenecks for large-scale production of BC^13^. To overcome these disadvantages and to improve BC production, three major strategies have been implemented: (1) identification of efficient and sustainable cellulose-producing strains^14^; (2) optimization of the fermentation process^12, 15^; and (3) improvement of productivity of the strains through genetic engineering. Strain mutagenesis is the most common approach to increasing BC production^16^. Up to a 2.3-fold increase in BC production was attained by knocking out the membrane-bound glucose dehydrogenase (GDH) enzyme responsible for oxidizing glucose to gluconic acid, which is the main by-product of BC fermentation^17^. Poor understanding of the detailed cellular metabolism involved in the production of BC prevents full exploitation of the industrial potential of *K. nataicola*. Therefore, the development of BC-synthesizing microbial cell factories would be worthwhile.

The advancement of the genome-sequencing technology stimulated research at the systemic level. As a result, genome-scale metabolic models (GSMMs) have been developed to characterize the physiological behavior and metabolic status of an organism subjected to different environmental and genetic changes^18, 19^. Advances in GSMMs have resulted in the identification of gene-protein-reaction associations, which have found broad applications in a number of processes, such as elucidation of physiological characteristics, organization of principles of metabolism, computational predictions for metabolic engineering, and identification of multi-species relations^20, 21^. More than 100 GSMMs for organisms across all three domains of life have been reconstructed so far^22^. For AABs, the only GSMM reported is *i*XW433 belonging to *Gluconobacter oxydans* 621H^23^. This model was used to examine the production of dihydroxyacetone by *G. oxydans* using glycerol as a carbon source. Nevertheless, no study so far has shown a refined GSMM for *K. nataicola*. The present study describes for the first time reconstruction of a GSMM for *K. nataicola* strain RZS01. The model, *i*HZ771, has been used to study cellulose production characteristics of AAB. Elucidation of the pathway for BC biosynthesis in strain RZS01 was based on genome annotation and literature mining. The model was also used to propose suitable strategies for improvement of BC production.

## Results and Discussion

### GSMM of *K. nataicola*

**Figure 1:**
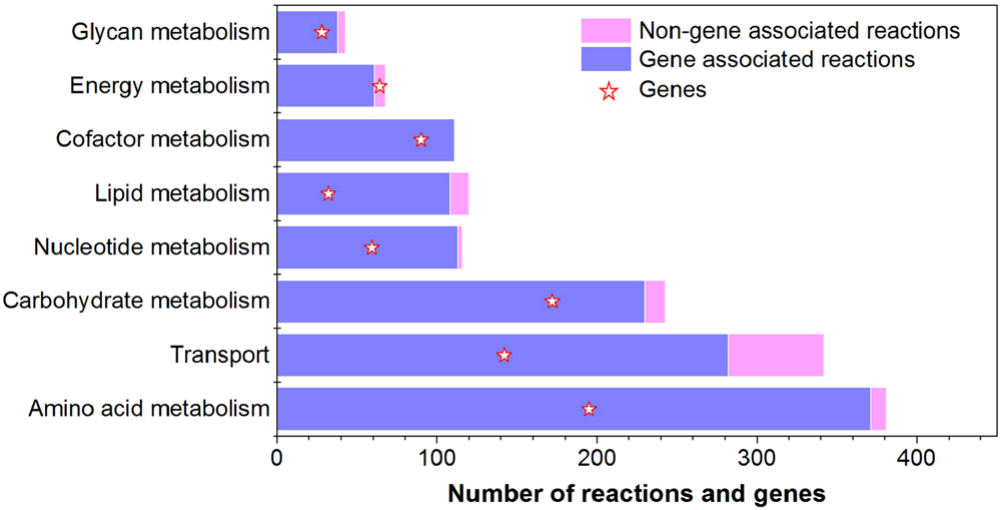
Distribution of genes and reactions across major metabolic sub-systems in *i*HZ771.

Reconstructed genome-scale model *i*HZ771 comprised 771 genes, 2035 metabolites, and 2014 reactions located throughout the cytosol, periplasm, and extracellular compartments, covering 21.9% of the annotated open reading frames. The metabolic reactions could be classified into eight major sub-systems: carbohydrates, amino acids, energy, cofactors, lipids, glycan, nucleotides, and transport^24^. Among these, the largest sub-system was amino acid metabolism (18.9%), followed by transport (17.0%), and carbohydrate metabolism (12.1%; Fig. 1). Complete information about the *i*HZ771 model, in terms of all the genes, reactions and metabolites is available as SBML in Supplementary Information 1.

**Figure 2:**
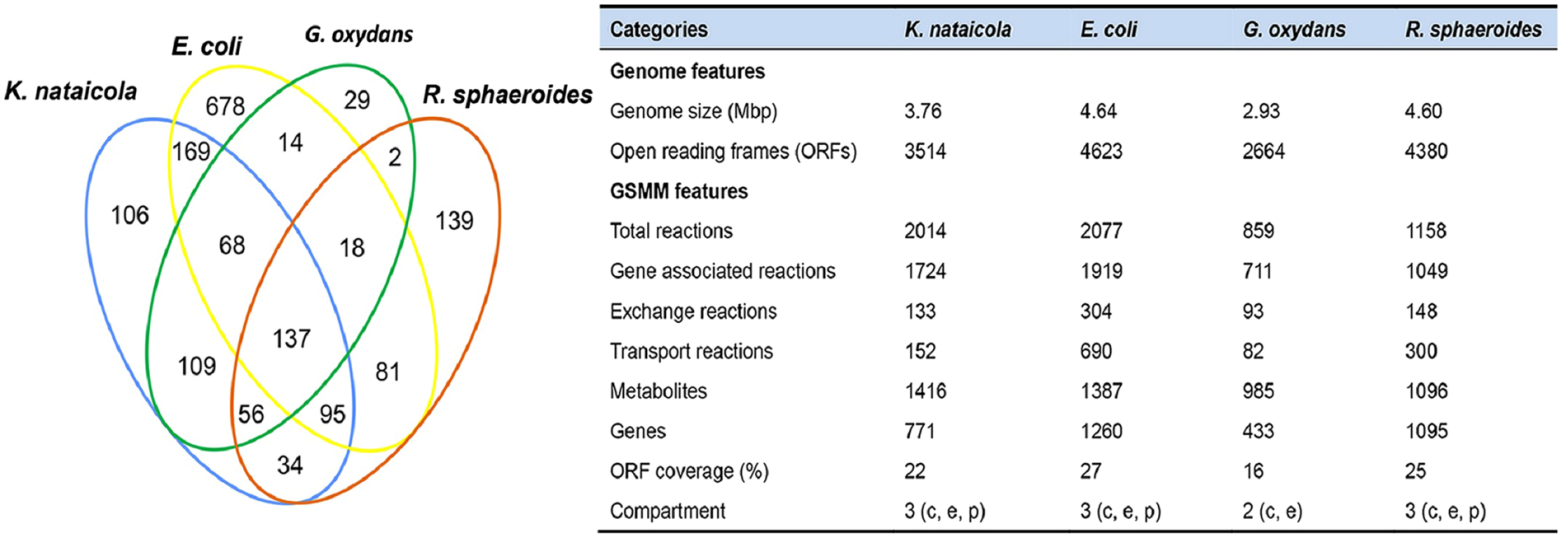
Comparison of general features of *i*HZ771 with *E. coli i*AF1260, *G. oxydans i*XW433, and *R. sphaeroides i*Rsp1095. Numerical values in each section of the Venn-diagram represent the number of genes that are common or specific to the respective organism.

Three related models were compared with model *i*HZ771 to understand its characteristics (Fig. 2). Coverage of the annotated ORFs in four models (*K. nataicola* RZS01, *E. coli* K-12 MG1655, *G. oxydans* 621 H, and *R. sphaeroides* 2.4.1) is 21.9%, 27.3%, 16.0%, and 25.0% respectively. A total of 137 genes, mostly belonging to central carbon metabolism (8.2%), amino acid metabolism (24.1%), and biosynthesis of the cytoskeleton (11.3%) were common among the models. The analysis also revealed that the *K. nataicola* model consists of 106 unique genes catalyzing 245 reactions, some of which belong to carbohydrate metabolism responsible for BC biosynthesis from glucose 6-phosphate. The analysis of transport reactions indicated that most of the amino acids and fatty acids are transported via ATP-binding cassette transporters or via co-transport, depending on the proton gradient; this situation results in excessive consumption of energy. Of note, in *K. nataicola*, conversion of ethanol to acetic acid is guided by a membrane-bound alcohol dehydrogenase, membrane-bound aldehyde dehydrogenase, and ubiquinol oxidase which generate a large amount of ATP at early stages^25^.

### Validation of the model

To assess the accuracy of model *i*HZ771, qualitative simulation of cellular growth in the presence of different carbon and nitrogen sources was carried out. Overall, 30 carbon sources (5 saccharides, 3 carboxylic acids, 2 alcohols, and 20 amino acids) and 25 nitrogen sources (ammonium, nitrate, urea, and 20 amino acids) were predicted for cell growth via flux balance analysis (FBA). Comparison of experimental data and the model prediction revealed that *K. nataicola* can utilize 15 types of carbon sources and 25 types of nitrogen sources (Supplementary Information 2-1, and 2-2). The precise (93%) match obtained showed that there were no serious faults in the model. The reasons for the discrepancies between the experiment and simulation include incomplete annotation and regulatory effects, which were not accounted for in model *i*HZ771^26^. Amino acids are biologically important small molecules, playing important roles in cell growth. *K. nataicola* is capable of synthesizing all the amino acids and does not require supplementation with amino acids^27^. On the other hand, many amino acids, such as proline, cysteine, and tyrosine, could not serve as carbon sources. The enzymes responsible for proline decomposition, including prolyl 4-hydroxylase (EC 1.14.11.2), 1-pyrroline dehydrogenase (EC 1.2.1.88), and 4-hydroxy-2-oxoglutaratealdolase (EC 4.1.3.16) are absent in *K. nataicola*. The incomplete decomposition pathway of proline shrinks the substrate utilization spectrum; glutamic acid and arginine are degraded to proline for further utilization^28^.

For quantitative assessment of the model’s accuracy at predicting the growth rate, we compared simulated growth phenotypes, obtained using glucose and ammonium as the sole carbon and nitrogen sources, respectively, with *in vivo* growth data^29^. The experimental data served as constraints to simulate cell growth parameters, including the glucose uptake rate, gluconic acid production rate, acetic acid production rate, and bacterial cellulose production rate. As shown in Supplementary Information 2–3, the growth rate *in silico* was highly consistent with the observed experimental data. Qualitative and quantitative assessments indicated that model *i*HZ771 may be sufficient to accurately describe the cellular metabolism of *K. nataicola*.

### Genes and reactions essential for cell growth

**Figure 3:**
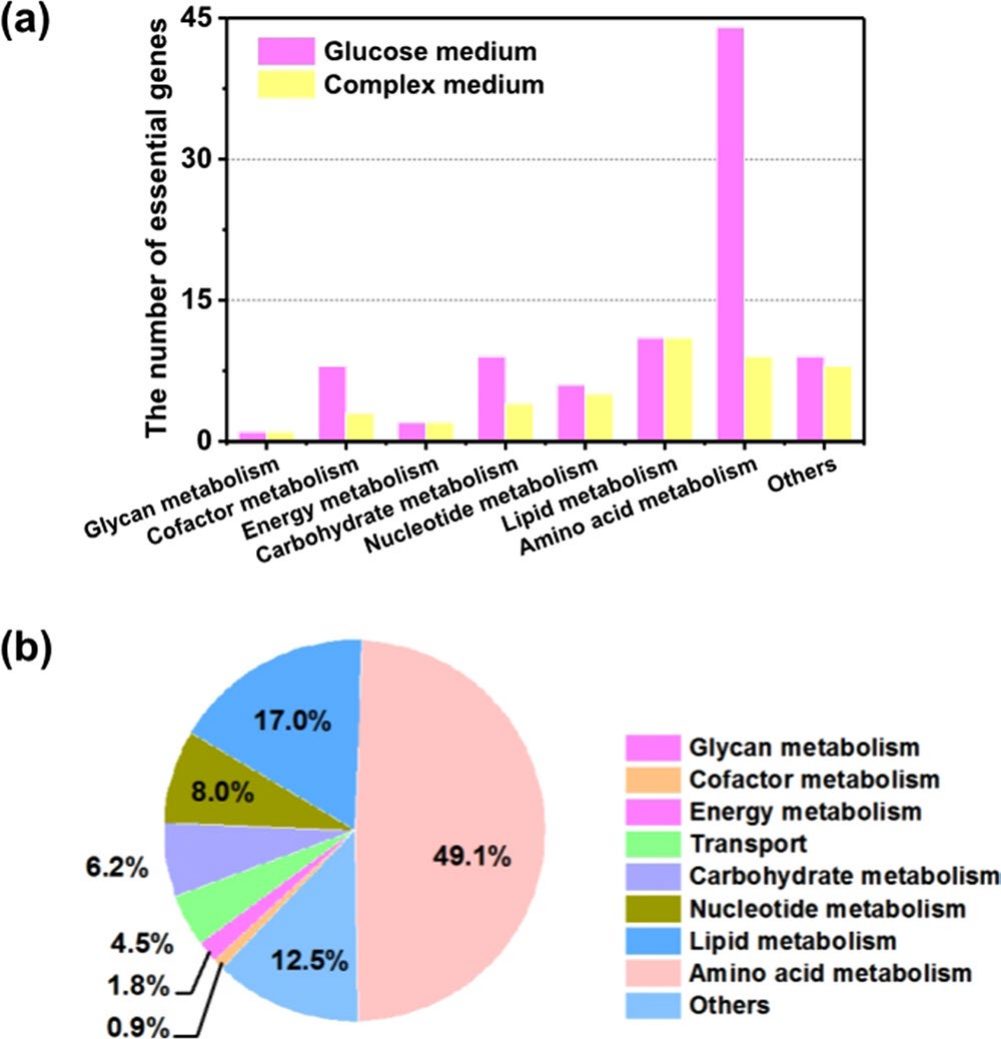
Distribution of essential genes and reactions in each subsystem. (**a)** The distribution of essential genes in a glucose-containing medium and a complex medium. **(b)** The percentage of essential reactions in each subsystem in a glucose-containing medium.

Analysis of essentiality of individual genes and reactions in *K. nataicola* was carried out in model *i*HZ771 using single gene deletion. It was found that 9.2% (71 genes) and 3.9% (30 genes) of the total of 771 genes in *i*HZ771 are necessary for cellular growth in a glucose-containing medium and complex medium, respectively. A total of 112 reactions were predicted to be essential for cell growth. We found that 68 reactions (60.7%) overlap with the corresponding reactions encoded by essential genes in a glucose-containing medium. The distribution of essential genes and reactions across various metabolic processes was also identified (Fig. 3). Most of the essential genes belong to the sub-systems of amino acid, lipid, and carbohydrate metabolic pathways. These genes encode proteins primarily involved in maintaining basic cellular structure and central metabolism. Furthermore, we found that all the genes necessary for growth in a complex medium are also required for growth in a glucose-containing medium. Genes identified in a glucose-containing medium mostly participate in amino acid biosynthesis or metabolism. For instance, glutamine synthetase encoded by *B0W47_07980* converts L-glutamate to L-glutamine, and *B0W47_14735* participates in the biosynthesis of L-arginine. Genes *B0W47_13090*, *B0W47_14725*, *B0W47_12370*, and *B0W47_13245* are crucial for the biosynthesis of L-lysine and L-threonine. In the complex medium, however, nutrient supplementation was required for these processes. The detailed lists of the essential genes under different conditions are provided in Supplementary Information 2–4.

### Broad utilization of different substrates

**Figure 4:**
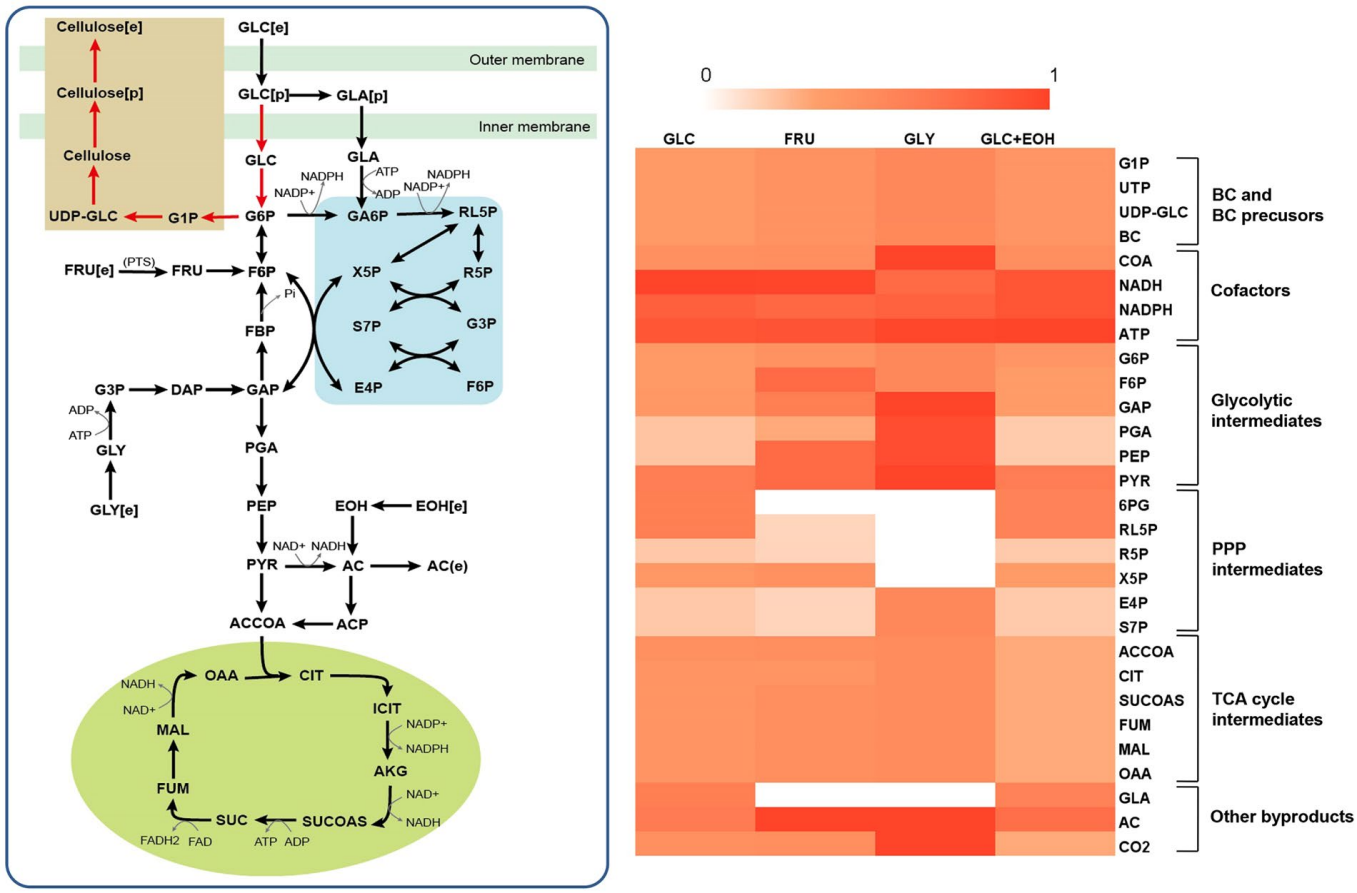
Flux-sum intensity comparisons for different carbon sources. A heatmap illustrating the flux-sum intensity of cofactors, by-products, and other components of central metabolism. The results were normalized to the maximal value of each metabolite, where the darker color indicates stronger flux.

Based on reconstructed model *i*HZ771, it was predicted that strain RZS01 can take up and channel several polyols, sugars, and sugar derivatives into the oxidative pentose phosphate pathway (PPP; Supplementary Information 2-5). Two main operative models exist for substrate transport, namely the phosphotransferase system (PTS) driving phosphorylation of substrates by utilizing phosphoenolpyruvate^30^, and ATP-binding cassette (ABC) transporters driven by ATP^31^. Constraint-based flux analysis simulations were carried out for *K. nataicola* grown on glucose, fructose, or glycerol, and the maximal uptake rate of each carbon source was set at −100 mmol/[(g of dry cell weight [DCW])·h] (negative sign indicates metabolite uptake into the cell; Fig. 4)^32^. As the most widely available carbon source, glucose was easily taken up by sugar permeases, encoded by *B0W47_14370*. The Embden-Meyerhof-Parnas pathway is incomplete in strain RZS01 because of a lack of the gene encoding phosphofructokinase (EC 2.7.1.11). Nevertheless, the genes encoding enzymes of the PPP were present, suggesting that glucose is degraded via the PPP, as also reported for *Acetobacter pasteurianus* 386B^4^ and *G. oxydans* 621H^33^. Besides, a large fraction of glucose is converted into gluconic acid in a reaction catalyzed by membrane-bound glucose dehydrogenase in the periplasm, resulting in a sharp decline in pH of the medium. It was also found that glycerol switches the pathway from PPP to the tricarboxylic acid (TCA) cycle, resulting in the oxidation of triose phosphate without formation of gluconic acid. This way, carbon is not wasted on CO_2_ production. Fructose has different metabolic fate, and is transported into the cell through PTS. This process is followed by degradation of pyruvate to acetate, followed by transformation to acetyl-CoA, which then enters the TCA cycle for energy synthesis. Addition of ethanol is believed to generate more of reduced nicotinamide adenine dinucleotide, which provides suitable environment for BC production by lowering the redox potential^34^. After comparison to the predicted results, we found that glycerol yielded the highest BC production, at 5.86 g/L, which was approximately 1.82-fold and 1.49-fold greater than BC production in the glucose and fructose medium, respectively. In the presence of ethanol, BC production was improved by 5.9%.

### *In silico* simulation of BC production

**Figure 5:**
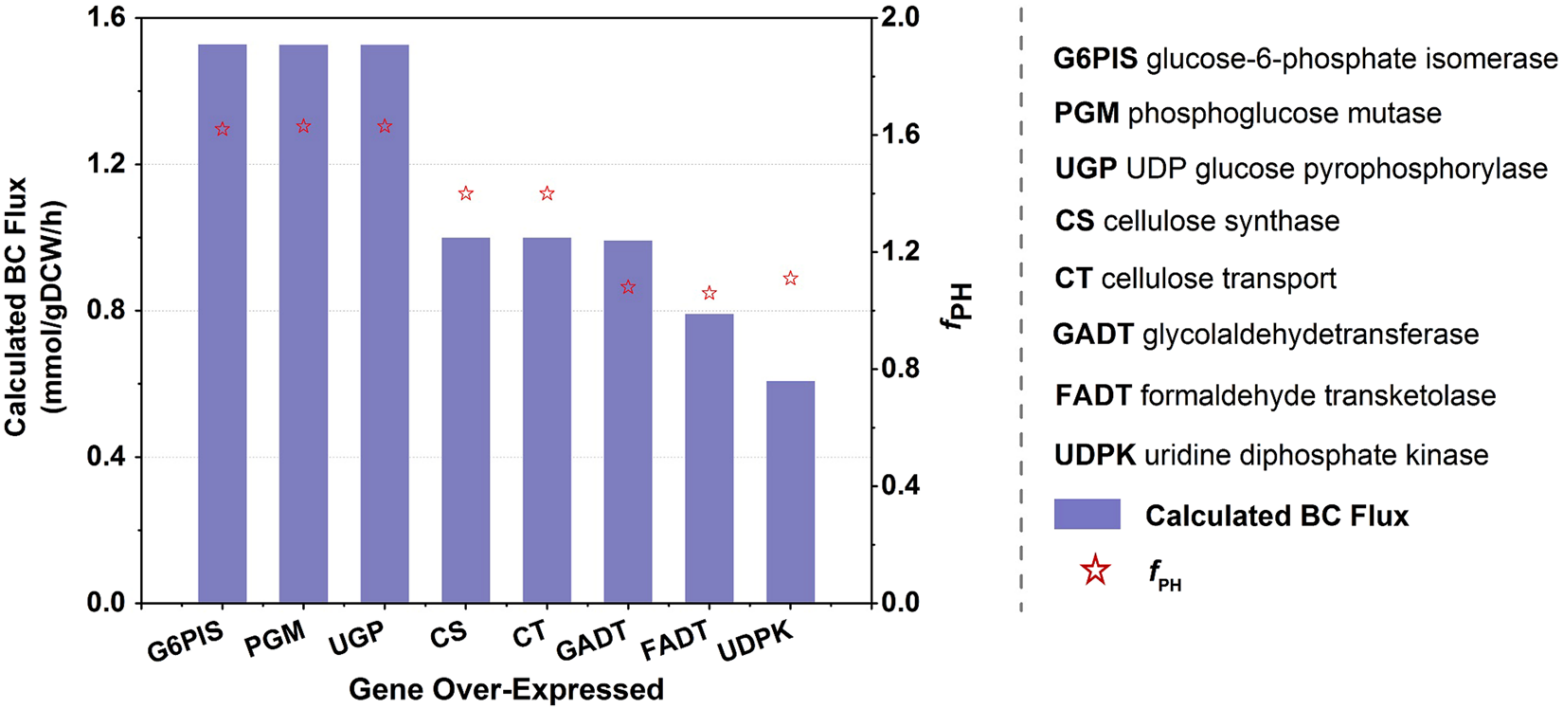
Calculated BC production flux and *f*_PH_ as a function of over-expression of a gene.

The BC biosynthetic pathway is independent of other metabolic pathways in the cell. Substrate synthesis for cellulose production processes starts from the glycolytic cycle intermediate glucose 6-phosphate. The first stage is isomerization of glucose 6-phosphate to glucose 1-phosphate, catalyzed by phosphoglucomutase (EC 5.4.2.2) encoded by *B0W47_02175* or *B0W47_13495*. This action is followed by the reaction of glucose 1-phosphate with UTP catalyzed by *B0W47_02365-* or *B0W47_02370-*encoded UTP-glucose-1-phosphate uridylyltransferase (EC 2.7.7.9), leading to the formation of uridine-5′-diphosphate-α-D-glucose (UDP-glucose). This is a rate-limiting step. Finally, the crucial enzyme, bacterial cellulose synthase (EC 2.4.1.12), transfers glucosyl residues from UDP-glucose to the nascent β-D-1,4-glucan chains. For identification of gene overexpression targets to achieve enhanced production of BC, minimization of metabolic adjustment (MOMA) was carried out to re-evaluate the fluxes for an over-expression algorithm. The experimental specific production rate of the wild type was found to be 0.5 mmol/[(g DCW)·h] and was set as the lower bound for BC transport flux. Under the given constraint conditions, the specific growth rate was found to be 0.3321 h^−1^ by FBA. Thereafter, all the reactions that have a non-zero flux value in the FBA simulation were over-expressed computationally. At the end of this process, 8 reactions were identified as the potential over-expression targets based on the *f*_PH_ value of greater than 1.0 (Fig. 5).

Of the eight targets identified, four (PGM, UGP, CS, and CT) are directly involved in the biosynthetic pathway of BC, whereas the remaining four targets (G6PIS, GADT, FADT, and UDPK) do not belong to the native BC precursor pathway. During the biosynthesis of BC, the membrane-integrated cellulose synthase (CS, EC: 2.4.1.12) containing BcsA, BcsB, BcsC, and BcsD, encoded by *B0W47_12635*, *B0W47_12640*, *B0W47_12645*, and *B0W47_12650*, respectively, is responsible for the formation and translocation of glucan chains. The activity of CS could be stimulated by its allosteric regulator, c-di-GMP, via PilZ domains^35^. Over-expression of CS led to enhanced BC production from 0.5 to 1.0 mmol/[(g DCW)·h]. As a mutase, PGM (EC: 5.4.2.2) catalyzes the conversion of D-glucose 6-phosphate into D-glucose 1-phosphate, which is the precursor of BC. A decrease in the growth rate by 47% was accompanied with a two-fold increase in BC production after over-expression of this gene. Bacterial cellulose biosynthesis may be regulated by energy metabolism. Both GADT (EC: 2.2.1.1) and FADT (EC: 2.2.1.2) participate in the PPP, which provides large amounts of NADPH for cellular biosynthesis and growth^25^. Over-expression of these genes led to 98% and 58% increases in BC production, respectively.

### Modelling of metabolic stressors

**Figure 6:**
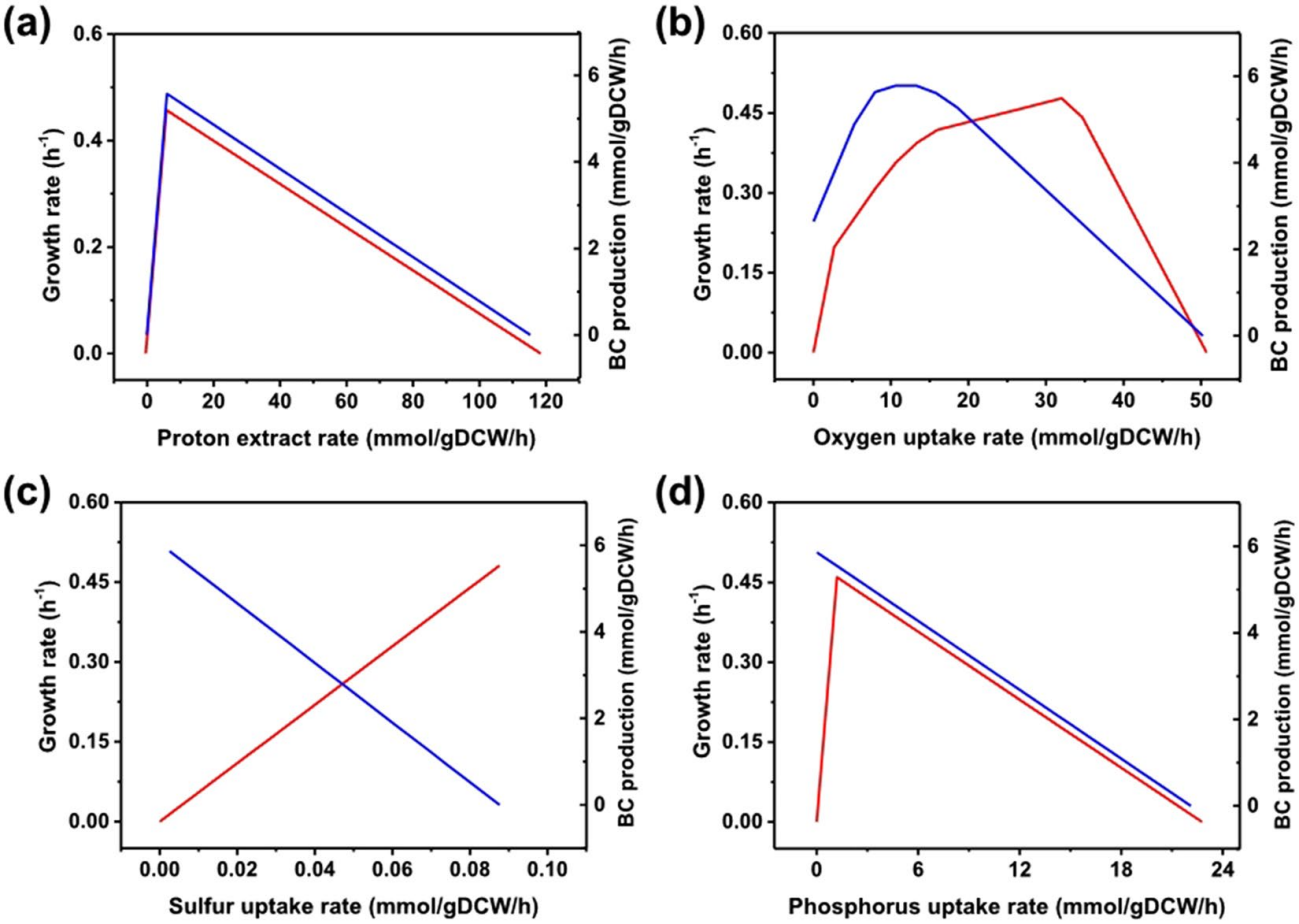
Simulation of the effects of perturbation of culture conditions on BC production and cell growth. Robustness analysis of the proton extraction rate **(a)**, oxygen uptake rate **(b)**, sulphur uptake rate **(c)**, and phosphorus uptake rate **(d)**. The red line indicates the cell growth rate, and the blue line denotes the BC production rate.

To further investigate the cellular behavior under the influence of changes in environmental conditions, robustness analysis was conducted. Cell growth and BC accumulation were found to possess similar adaptive capacity (Fig. 6). Simulation data revealed that cell growth and BC production are both resistant to high absorption of protons, in agreement with the fact that this strain was initially isolated from vinegar brew^36^. Nonetheless, increasing the proton extraction rate over 12 mmol/[(g DCW)·h] resulted in inhibition of cell growth and of BC accumulation. In strict aerobic bacteria, oxygen plays a crucial role in maintaining intracellular metabolism, being the primary electron acceptor^37^. According to simulation intended to predict the phenotype at different oxygen uptake rates, the highest BC production was observed at the oxygen uptake rate of 10 mmol/[(g DCW)·h]. For cellular growth, 32 mmol/[(g DCW)·h] was found to be the best uptake rate, which was far above that for BC accumulation. These simulation results, namely, that higher oxygen tension inhibits cellulose production, are in line with the results obtained by Hwang^38^. We, therefore, determined that a relatively high concentration of dissolved oxygen is beneficial for cell proliferation at the first stage. Nevertheless, to avoid the excessive oxygen exposure, a low dissolved-oxygen concentration is employed during BC production.

As for other environmental disturbances, such as changes in sulphur and phosphate content, robustness analysis was carried out. Sulphur and phosphate perform key functions in the biosynthesis of the cell scaffold, where the former participates in the biosynthesis of sulphur-containing amino acids: methionine and cysteine. The simulation results indicated that a high sulphur uptake rate is beneficial for cell growth, resulting in the flux of a large amount of substrates to biomass. This finding is in agreement with other reports^39^. Phosphate is mainly present in plasma membrane, nucleic acids, and some coenzymes. Nonetheless, when the uptake rate of phosphate reaches 1.25 mmol/[(g DCW)·h], cell growth is retarded. This prediction is supported by one study, where a decrease in BC production was obtained after supplementation with phosphate sources^40^.

## Conclusions

Our study describes a GSMM, *i*HZ771, for *K. nataicola*. Validation of the model revealed that it can accurately simulate phenotypic behavior under various conditions. Analysis of essentiality of genes and reactions highlighted the importance of the metabolic backbone for cell growth. Flux intensity analysis in the presence of different carbon sources revealed that glycerol is beneficial for BC production. Eight simulated targets responsible for increasing BC production were identified via the MOMA algorithm. The lack of experimental information on gene over-expression is still a setback for the validation of the model created here. Further experiments on this should help us better refine and complete the networks. Overall, the proposed model is expected to be useful for systemic analysis of AAB and should facilitate future biotechnological applications.

## Methods

### Whole-genome sequencing

Genomic-DNA isolation and purification from *K. nataicola* RZS01 was carried out as per the protocol described by Florea *et al*.^41^. Quality of the genomic DNA was assessed by agarose gel electrophoresis. Whole-genome sequencing was carried out using an Illumina technology (HangZhou GeneRui Biotechnology Co. Ltd., Hangzhou, China). As a consequence, the complete genomic sequence, composed of a circular chromosome and six plasmids, was obtained. The accession number of the complete sequences of this strain from this study can be found in GenBank (http://www.ncbi.nlm.nih.gov) under the accession no. CP019875 to CP019881.

### Reconstruction and refinement of the GSMM

The general principles of GSMM reconstruction process have been described elsewhere^42^. Whole-genome sequence of *K. nataicola* RZS01was uploaded to the RAST server (http://rast.nmpdr.org/) to call and annotate the genes. The initial model was constructed based on the protein homology through a local sequence similarity search (BLASTp). Three existing models, *i*XW433, *i*Rsp1095, and *i*AF1260 belonging to *Gluconobacter oxydans* 621 H, *Rhodobacter sphaeroides* 2.4.1, and *Escherichia coli* K-12 MG1655^43^, respectively, served as templates. The homology search parameters for prokaryotes in BLASTp were set at an identity of ≥35%, and an *e*-value of ≤10^−6^ ^44^. The reaction lists from BLASTp were integrated in the same format. This action was followed by manual refinement of the model with the help of public databases, such as KEGG, MetaCyc, Biopath, CELLO, and the COBRA software package. The process involved the following steps: (1) assignment of appropriate subcellular compartments; (2) determination of reaction directionality; (3) addition of metabolite transport and exchange reactions; (4) checking the consistency in terms of element and charge balance; (5) verifying connectivity of the network by the GapFind algorithm followed by filling or correcting the reactions from other organisms according to the literature.

### Biomass composition

An equation, based on experimental analysis and literature that describes the cellular composition is widely used as an objective function in the constraint-based model. The major macromolecular constituents of the cells in the exponential growth phase were quantified to generate an appropriate biomass equation. These components consisted of DNA, RNA, proteins, lipids, cell wall constituents, and small molecules. DNA and RNA were quantified using a mini kit. G + C content (61.49%) was used to determine the individual weights of nucleotides in DNA and RNA. Coomassie brilliant blue staining was carried out to determine protein content^45^. Because the components of amino acids were not available, we utilized the genome information to estimate the amino acid composition. Lipid analysis was conducted as described previously^46^. As a member of family *Acetobacteriaceae*, strain *G. oxydans* 621 H was subjected to the analysis of biomass composition, specifically composition of peptidoglycan^23^. In all the simulations carried out in the present study, biomass composition was assumed to be constant under different environmental conditions. Growth-associated maintenance energy requirement was estimated according to the protocol described for *E. coli* ^43^. Detailed information on calculation of biomass composition is provided in Supplementary Information 3.

### *In vivo* growth screens

Growth screens were carried out in triplicate in a 250-mL baffled-flask containing 50 mL of culture broth. For qualitative simulation of cellular growth in the presence of different carbon and nitrogen sources, a defined medium was used for analysis, which contained 20 g of a carbon source, 5 g of a nitrogen source, 3 g of Na_2_HPO_4_, 1 g of KH_2_PO_4_, 0.02 g of MgCl_2_, 0.02 g of CaCl_2_, and 0.0015 g of aminobenzoic acid per litre, and the initial pH was adjusted to 6.0. The medium used for seed culture contained 20 g of glucose, 6 g of (NH_4_)_2_SO_4_, 1 g of KH_2_PO_4_, 0.4 g of MgSO_4_, 3 g of peptone, 2.25 g of yeast extract, and 0.4 g of sodium carboxymethylcellulose per litre. To prepare for the fermentation process, the strain was cultured in a seed medium for 36 h. Next, the seed cultures were centrifuged for 10 min at 8,000 × g, and then washed twice with a 0.9% NaCl solution. After that, the supernatant was decanted and resulting pellet was resuspended in 50 mL of the NaCl solution, of which 4 mL (equivalent to 8% of the inocula) was inoculated into 50 mL of the culture medium in 250-mL flasks. The cultures were grown at 30 °C, with shaking at 160 rpm for 5 days.

### Analytical methods

Concentrations of glucose, organic acids and ethanol were measured by means of a high-performance liquid chromatography (HPLC) system (Agilent, USA) equipped with an HPX-87H column (Bio-Rad, Hercules, CA) and a dual λ absorbance detector. To determine DCW, the culture broth was resuspended in 0.2% (w/v) cellulase in citric acid-sodium citrate buffer (pH 4.8) and hydrolyzed for 2 h at 50 °C, then filtered through filter paper. The cells were dried overnight in an oven and weighed on filter paper, with a correction for the amount of weight lost by the filter paper that did not contain cells. The BC produced in the shake flask was collected from the medium directly and treated with 4% (w/v) NaOH at 85 °C for 2 h to remove impurities and cells attached to cellulose. Then, the BC was washed in tap water until the pH of water became neutral. The purified BC was dried to constant weight at 80 °C and weighed. Growth rates were calculated by determining the exponential growth phase region in a series of samples taken during each growth screen. For this corresponding exponential growth phase region, the ratio of an analyte to grams of dry weight (DW) of the sample was determined using a linear fit obtained by the least-squares method (‘regress’ function in MATLAB). This value (mmol/g DW) was then multiplied by the growth rate to obtain the corresponding uptake or production rate. The ratios were finally multiplied by the growth rate to obtain the uptake or production rates.

### Constraint-based flux analysis

This analysis was used to simulate cellular metabolism of *K. nataicola* under varying environmental conditions. The analysis was conducted by means of Cobra Toolbox 2.05 with MATLAB 2012b and Gurobi 6.5.1 optimizer. Identification of the over-expression targets was carried out using the MOMA framework for better prediction of the flux distribution. The over-expression algorithm involved five steps^47^: (1) BC production flux (as determined experimentally) was imposed upon the reconstructed model; (2) flux for each reaction was calculated based on the complex medium by adjusting uptake rates of specific chemical components of the medium, such as the basic elements and 20 amino acids. The maximal rates of uptake of amino acids were set to 0.1 mmol/[(g DCW)·h]. Glucose served as the sole carbon source, and its uptake rate was set to 8.45 mmol/[(g DCW)·h], as described elsewhere^29^; (3) amplifying flux was imposed upon individual reactions with non-zero flux (to simulate the effect of gene over-expression); (4) MOMA was performed to solve the problem of over-expression; (5) finally, identification of the over-expression targets was done and led to a phenotype fraction value, *f*_PH_, greater than unity [equation (1)]. Steps 3 and 4 were iterated for every reaction inside the model^24^.

1 $${f}_{{\rm{PH}}}=({{\rm{f}}}_{{\rm{biomass}}})\,{({\rm{f}}}_{{\rm{BC}}})=(\frac{{{\rm{V}}}_{{\rm{biomass}},{\rm{over}}-{\rm{expression}}}}{{{\rm{V}}}_{{\rm{biomass}},{\rm{wide}}}})(\frac{{{\rm{V}}}_{{\rm{BC}},{\rm{over}}-{\rm{expression}}}}{{{\rm{V}}}_{{\rm{BC}},{\rm{wide}}}})$$

### Flux-sum

Constraint-based flux analysis indicates only the reaction rates in terms of fluxes, and does not provide any information about the concentration of metabolites. Therefore, the concept of ‘flux-sum’ (*ϕ*_*i*_) was incorporated into then model to compare the turnover rates of metabolites in *Komagataeibacter*. The flux-sum of metabolite *i* can mathematically be formulated as equation (2):

2 $$\,{{\rm{\varphi }}}_{{\rm{i}}}=\sum _{{{\rm{j}}{\rm{\varepsilon }}{\rm{P}}}_{{\rm{i}}}}{{\rm{S}}}_{{\rm{ij}}}{{\rm{v}}}_{{\rm{j}}}=-\sum _{{{\rm{j}}{\rm{\varepsilon }}{\rm{C}}}_{{\rm{i}}}}{{\rm{S}}}_{{\rm{ij}}}{{\rm{v}}}_{{\rm{j}}}=\frac{1}{2}{\sum }^{}|{{\rm{S}}}_{{\rm{ij}}}{{\rm{v}}}_{{\rm{j}}}|$$

where *S*_*ij*_ is the stoichiometric coefficient of metabolite *i* involved in reaction *j*, and *v*_*j*_ is flux or specific rate of metabolic reaction *j*. *P*_*i*_ denotes the set of reactions producing metabolite *i*, and *C*_*i*_ represents the set of reactions consuming metabolite *i* ^48^.

## Electronic supplementary material

Supplementary Information 1

Supplementary Information 2

Supplementary Information 3
