# Supplementary material for: Reconstruction of a Genome-scale Metabolic Network of Komagataeibacter nataicola RZS01 for Cellulose Production
Source: Sci Rep. 2017 Aug 11;7:7911. doi: 10.1038/s41598-017-06918-1 (PMC5554229; doi:10.1038/s41598-017-06918-1)
Supplement: Supplementary file 2 — Supplementary Information 2 [file 41598_2017_6918_MOESM2_ESM.doc]

Supplementary Information 2

**Reconstruction of** **a Genome-scale Metabolic Network of** [***Komagataeibacter***](http://www.ncbi.nlm.nih.gov/genome/2728) ***nataicola* RZS01 for Cellulose Production**

**Heng Zhang**^1,2^**, Chao Ye**^3^**, Nan Xu**^3^**, Chuntao Chen**^1,2^**, Xiao Chen**^1,2^**, Fanshu Yuan**^1,2^**, Yunhua Xu**^4^**, Jiazhi Yang**^1,2✻^**& Dongping Sun**^1,2†^

^1^ Chemicobiology and Functional Materials Institute, Nanjing University of Science and Technology, Nanjing, 210094, China

^2^ School of Chemical Engineering, Nanjing University of Science and Technology, Nanjing, 210094, China

^3^ State Key Laboratory of Food Science and Technology, Jiangnan University, Wuxi, 214122, China

^4^ Department of Life Sciences, Lianyungang Normal College, Lianyungang, 222000, China

^✻^Corresponding author, Jiazhi Yang, e-mail: jiazhiyang@sina.com

^†^ Corresponding author: Dongping Sun, e-mail: sundpe301@163.com

**Supplementary Information 2-1: Validation of growth phenotype under a sole carbon source.**

| **Substrate** |  | **Biomass** | | **References** |
| --- | --- | --- | --- | --- |
| **Carbon sources** |  | ***in vivo*** | ***in silico*** |  |
| Saccharides | Glucose | + | + | This study |
|  | Galactose | + | + | [1] |
|  | Sucrose | + | + | This study |
|  | Starch | - | - | This study |
|  | Glycerol | + | + | This study |
| Carboxylic acids | Gluconate | + | + | This study |
|  | Acetate | + | + | This study |
|  | Malate | + | + | This study |
| Alcohols | Sorbitol | + | + | This study |
|  | Mannitol | + | + | [2] |
| Amino acids | L-alanine | + | + | This study |
|  | L-glutamic acid | - | - | This study |
|  | L-glutamine | - | - | This study |
|  | L-glycine | - | + | This study |
|  | L-threonine | + | + | This study |
|  | L-aspartic acid | + | + | This study |
|  | L-asparagine | + | + | This study |
|  | L-tryptophan | + | - | This study |
|  | L-histidine | - | - | This study |
|  | L-serine | + | + | This study |
|  | L-tyrosine | - | - | This study |
|  | L-valine | - | - | This study |
|  | L-lysine | - | - | This study |
|  | L-arginine | - | - | This study |
|  | L-cysteine | - | - | This study |
|  | L-proline | - | - | This study |
|  | L-phenylalanine | - | - | This study |
|  | L-leucine | - | - | This study |
|  | L-isoleucine | - | - | This study |
|  | L-methionine | - | - | This study |

**Supplementary Information 2-2: Validation of growth phenotype under a sole nitrogen source.**

| **Substrate** |  | **Biomass** | | **References** |
| --- | --- | --- | --- | --- |
| **Nitrogen sources** |  | ***in vivo*** | ***in silico*** |  |
|  | (NH_4_)_2_SO_4_ | + | + | This study |
|  | Ammonia | + | + | This study |
|  | (NH_4_)_2_HPO_4_ | + | + | This study |
|  | NaNO_3_ | + | + | This study |
|  | Urea | + | + | This study |
| Amino acids | L-alanine | + | + | This study |
|  | L-glutamic acid | + | + | This study |
|  | L-glutamine | + | + | This study |
|  | L-glycine | + | + | This study |
|  | L-threonine | + | + | This study |
|  | L-aspartic acid | + | + | This study |
|  | L-asparagine | + | + | This study |
|  | L-tryptophan | - | + | This study |
|  | L-histidine | + | + | This study |
|  | L-serine | + | + | This study |
|  | L-tyrosine | + | + | This study |
|  | L-valine | + | + | This study |
|  | L-lysine | + | + | This study |
|  | L-arginine | + | + | This study |
|  | L-cysteine | + | + | This study |
|  | L-proline | + | + | This study |
|  | L-phenylalanine | + | + | This study |
|  | L-leucine | + | + | This study |
|  | L-isoleucine | + | + | This study |
|  | L-methionine | + | + | This study |

**Supplementary Information 2-3: Comparison between experimental data and simulation prediction.**

| **Constraints (mmol/gDCW/h)** | | | |  | **Growth rate (h^-1^)** | |
| --- | --- | --- | --- | --- | --- | --- |
| **GUR** | **GAPR** | **HAPR** | **BCPR** |  | ***In vivo*** | ***In silico*** |
| 8.45 | 4.94 | 0.8 | 2.05 |  | 0.014 | 0.0144 |

**Supplementary Information 2-4: Essential genes under different conditions.**

| **Glucose-containing media** | **Complex media** |
| --- | --- |
| peg.1079 | peg.1330 |
| peg.1247 | peg.1369 |
| peg.1307 | peg.1617 |
| peg.1310 | peg.1651 |
| peg.1311 | peg.1785 |
| peg.1330 | peg.1846 |
| peg.1349 | peg.1860 |
| peg.1369 | peg.1918 |
| peg.1371 | peg.1919 |
| peg.1372 | peg.1970 |
| peg.1440 | peg.2033 |
| peg.1585 | peg.2239 |
| peg.1617 | peg.2329 |
| peg.1651 | peg.2428 |
| peg.1721 | peg.2516 |
| peg.1785 | peg.2565 |
| peg.1804 | peg.2575 |
| peg.1846 | peg.2608 |
| peg.1860 | peg.2645 |
| peg.1889 | peg.2661 |
| peg.1918 | peg.295 |
| peg.1919 | peg.2979 |
| peg.1920 | peg.2981 |
| peg.1927 | peg.3025 |
| peg.1970 | peg.492 |
| peg.2005 | peg.500 |
| peg.2033 | peg.543 |
| peg.2239 | peg.712 |
| peg.2284 | peg.717 |
| peg.2329 | peg.727 |
| peg.2428 |  |
| peg.2436 |  |
| peg.2471 |  |
| peg.2516 |  |
| peg.2517 |  |
| peg.2553 |  |
| peg.2565 |  |
| peg.2572 |  |
| peg.2575 |  |
| peg.2608 |  |
| peg.2618 |  |
| peg.2619 |  |
| peg.2645 |  |
| peg.2652 |  |
| peg.2661 |  |
| peg.295 |  |
| peg.2952 |  |
| peg.2955 |  |
| peg.2958 |  |
| peg.2979 |  |
| peg.2981 |  |
| peg.3025 |  |
| peg.470 |  |
| peg.492 |  |
| peg.500 |  |
| peg.501 |  |
| peg.543 |  |
| peg.575 |  |
| peg.595 |  |
| peg.617 |  |
| peg.619 |  |
| peg.62 |  |
| peg.707 |  |
| peg.708 |  |
| peg.712 |  |
| peg.717 |  |
| peg.727 |  |
| peg.859 |  |
| peg.881 |  |
| peg.897 |  |
| peg.977 |  |

**Supplementary Information 2-5: The pathways for the utilization of different substrates in *K. nataicola* RZS01.**

**
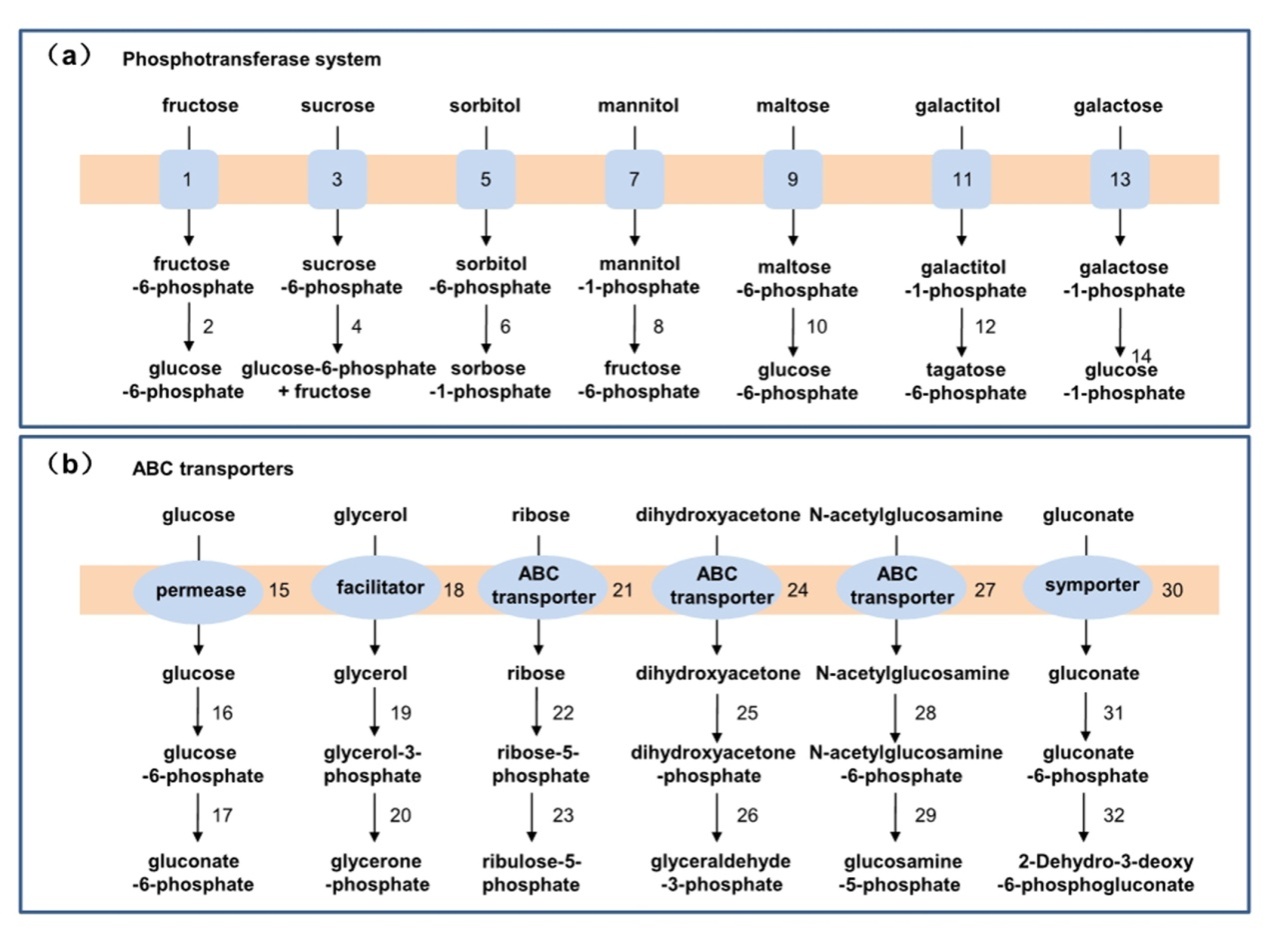
**

The abbreviations of each enzyme: (1) phosphoenolpyruvate-protein phosphotransferase; (2) glucose-6-phosphate isomerase; (3) phosphoenolpyruvate-

protein phosphotransferase; (4) levansucrase; (5) phosphoenolpyruvate-protein phosphotransferase; (6) mannonate oxidoreductase; (7) phosphoenolpyruvate-protein phosphotransferasel; (8) mannitol-1-phosphate-5-dehydrogenase; (9) phosphoenolpyruvate-protein phosphotransferasel; (10) maltose-6-phosphate glucosidase; (11) phosphoenolpyruvate-protein phosphotransferasel; (12) galactitol-1-phosphate dehydrogenase; (13) galactose phosphotransferase; (14) uridyltransferase; (15) glucose permease; (16) glucokinase; (17) hexose-6-phosphate dehydrogenase; (18) glycerol uptake facilitator protein; (19) glycerol kinase; (20) glycerol-3-phosphate dehydrogenase; (21) ribose ABC transporter; (22) ribokinase; (23) ribose-5-phosphate isomerase; (24) dihydroxyacetone ABC transporter; (25) dihydroxyacetone kinase; (26) triose-phosphate isomerase; (27) N-acetylgylucosamine permease; (28) N-acetylgylucosamine kinase; (29) N-acetylgylucosamine-6-phosphate deacylase; (30) gluconate symporter; (31) gluconokinase; (32) 6-phosphogluconate dehydratase.

**References**

[1] Fang, L. & Catchmark, J. M. Characterization of cellulose and other exopolysaccharides produced from Gluconacetobacter strains. *Carbohydr. Polym*. **115**, 663-669 (2005).

[2] Mikkelsen, D., Flanagan, B. M., Dykes, G. A. & Gidley, M. J. Influence of different carbon sources on bacterial cellulose production by Gluconacetobacter xylinus strain ATCC 53524. *J. Appl. Microbiol.* **107**, 576-583 (2009).
