# Supplementary material for: Reconstruction of a Genome-scale Metabolic Network of Komagataeibacter nataicola RZS01 for Cellulose Production
Source: Sci Rep. 2017 Aug 11;7:7911. doi: 10.1038/s41598-017-06918-1 (PMC5554229; doi:10.1038/s41598-017-06918-1)
Supplement: Supplementary file 3 — Supplementary Information 3 [file 41598_2017_6918_MOESM3_ESM.doc]

Supplementary Information 3

**Reconstruction of** **a Genome-scale Metabolic Network of** [***Komagataeibacter***](http://www.ncbi.nlm.nih.gov/genome/2728) ***nataicola* RZS01 for Cellulose Production**

**Heng Zhang**^1,2^**, Chao Ye**^3^**, Nan Xu**^3^**, Chuntao Chen**^1,2^**, Xiao Chen**^1,2^**, Fanshu Yuan**^1,2^**, Yunhua Xu**^4^**, Jiazhi Yang**^1,2✻^**& Dongping Sun**^1,2†^

^1^ Chemicobiology and Functional Materials Institute, Nanjing University of Science and Technology, Nanjing, 210094, China

^2^ School of Chemical Engineering, Nanjing University of Science and Technology, Nanjing, 210094, China

^3^ State Key Laboratory of Food Science and Technology, Jiangnan University, Wuxi, 214122, China

^4^ Department of Life Sciences, Lianyungang Normal College, Lianyungang, 222000, China

^✻^Corresponding author, Jiazhi Yang, e-mail: jiazhiyang@sina.com

^†^ Corresponding author: Dongping Sun, e-mail: sundpe301@163.com

**Supplementary Information 3:** **Biomass composition of *K. nataicola* RZS01**

**Biomass equation**: 0.5600AlaM00042[c] + 0.3399ArgM00061[c] + 0.1197Asn[c] + 0.2512Asp[c] + 0.0493Cys[c] + 0.1575Gln[c] + 0.2221Glu[c] + 0.3879Gly[c] + 0.1163His[c] + 0.2209Ile[c] + 0.4476Leu[c] + 0.1172Lys[c] + 0.1328Met[c] + 0.1514Phe[c] + 0.2604Pro[c] + 0.2340Ser + 0.2594Thr + 0.0629Trp + 0.1019Tyr + 0.3243Val + 0.0045dAMP+0.0045dTMP+0.0072dGMP+0.0072dCMP + 0.0904AMP[c] + 0.1218GMP[c] + 0.1172CMP[c] + 0.0863UMP[c] + 0.0360 PC[c] + 0.0333 CL[c] + 0.0289 PE[c] + 0.0044 PG[c] + 0.0324 Hexadecanoic acid[c] + 0.0762 Octadecenoic acid[c] + 0.0063 Octadecanoic acid[c] + 0.0413 Glycerol[c] + 0.0660 NAG[c] + 0.0498 NAM[c] + 1.0824 Carbohydrates[c] → biomass[c]

**Table 1 Macromolecular composition**

A complete cellular composition of *Komagataeibacter nataicola* RZS01.

| **Component** | **Cellular content (g/g DCW)** | **Reference** |
| --- | --- | --- |
| **DNA** | 0.0071 | In this study |
| **RNA** | 0.1340 |
| **Protein** | 0.5716 |
| **Lipids** | 0.0736 | [1][2][3] |
| Phospholipids | 0.0386 |
| phosphatidylcholine | 0.0099 |
| cardiolipin | 0.0166 |
| phosphatidylethanolamine | 0.0078 |
| phosphatidylglycerol | 0.0040 |
| Fatty acids and glycerol | 0.0350 |
| Hexadecanoic acid | 0.0083 |
| Octadecenoic acid | 0.0214 |
| Octadecanoic acid | 0.0018 |
| Glycerol | 0.0038 |
| **Peptidoglycan** | 0.0292 | [4] |
| NAG | 0.0146 |
| NAM | 0.0146 |
| **Carbohydrates** | 0.1752 |
| **Ash** | 0.0093 |
| Sum | 1.0000 |  |

**Table 2 Protein composition**

| **amino acids** | **abbreviation** | **Count (all ORFs)** | **Prevalence (%)** | **MW^a^, g/mol** | **P*MW (%)** | **by weight (%)** | **mmol/g DCW** |
| --- | --- | --- | --- | --- | --- | --- | --- |
| Alanine | Ala (A) | 128819 | 12.39874568 | 89.09 | 1104.653847 | 8.728681525 | 0.560005653 |
| Arginine | Arg (R) | 78188 | 7.525544579 | 174.20 | 1310.972442 | 10.3589563 | 0.339901117 |
| Asparagine | Asn (N) | 27536 | 2.650322243 | 132.12 | 350.1579244 | 2.766854986 | 0.119705289 |
| Aspartate | Asp (D) | 57787 | 5.561961485 | 133.10 | 740.3193215 | 5.849806796 | 0.251213304 |
| Cysteine | Cys (C) | 11340 | 1.091467687 | 121.16 | 132.242225 | 1.044942964 | 0.049297573 |
| Glutamate | Gln (Q) | 36232 | 3.487306635 | 146.15 | 509.6559155 | 4.02716578 | 0.15750879 |
| Glutamine | Glu (E) | 51079 | 4.916320811 | 147.13 | 723.3431972 | 5.715665967 | 0.222052094 |
| Glycine | Gly (G) | 89231 | 8.588426208 | 75.07 | 644.7073901 | 5.09430669 | 0.387907563 |
| Histidine | His (H) | 26760 | 2.575632743 | 155.16 | 399.6248739 | 3.157729692 | 0.11633184 |
| Isoleucine | Ile (I) | 50812 | 4.890622233 | 131.18 | 641.5273714 | 5.069179026 | 0.220891384 |
| Leucine | Leu (L) | 102962 | 9.910026103 | 131.18 | 1299.947674 | 10.27184151 | 0.447599361 |
| Lysine | Lys (K) | 26970 | 2.595845108 | 146.19 | 379.4840005 | 2.998581856 | 0.117244758 |
| Methionine | Met (M) | 30554 | 2.940802797 | 149.21 | 438.8089485 | 3.46735185 | 0.132825225 |
| Phenylalanine | Phe (F) | 34816 | 3.351017548 | 165.19 | 553.5612908 | 4.374094403 | 0.151353114 |
| Proline | Pro (P) | 59891 | 5.764470128 | 115.13 | 663.6749748 | 5.24418351 | 0.260359874 |
| Serine | Ser (S) | 53820 | 5.180140293 | 105.09 | 544.3964838 | 4.301676531 | 0.233967848 |
| Threonine | Thr (T) | 59663 | 5.742525275 | 119.12 | 684.0496108 | 5.405178476 | 0.259368705 |
| Tryptophan | Trp (W) | 14470 | 1.392728169 | 204.23 | 284.4340884 | 2.247522677 | 0.062904399 |
| Tyrosine | Tyr (Y) | 23437 | 2.255796136 | 181.19 | 408.7299577 | 3.229675648 | 0.101885999 |
| Valine | Val (V) | 74601 | 7.180298142 | 117.15 | 841.1575667 | 6.646603847 | 0.324307608 |
| sum |  | 1038968.0000 | 100.0000 |  | 12655.4491 | 100.0000 | 4.5166 |

The amino acids were identified in this study.

**Table 3 DNA composition**

The composition of DNA was based on the genomic sequence of *Komagataeibacter nataicola* RZS01.

G+C content (%): 61.49

| **content** | **mol/mol DNA** | **MW(g/mol)** | **mmol/g DNA** | **mmol/g DCW** |
| --- | --- | --- | --- | --- |
| dAMP | 0.1926 | 331.2000 | 0.6319 | 0.0045 |
| dGMP | 0.3074 | 304.2000 | 1.0086 | 0.0072 |
| dCMP | 0.3074 | 289.2000 | 1.0086 | 0.0072 |
| dTMP | 0.1926 | 304.2000 | 0.6319 | 0.0045 |
| average | 1.0000 | 304.7892 |  | 0.0234 |

**Table 4 RNA composition**

It was assumed that RNA consisted of 5% mRNA, 75% rRNA and 20% tRNA (molar). The nucleotide composition of mRNA was taken as for genomic DNA. The nucleotide composition of rRNA was calculated from the sequences of 16S, 23S and 5S ribosomal RNA units. tRNA composition was found from sequences of amino acids transporting RNAs. All the sequences were obtained from GenBank.

| **Nucleotide** | **mol/mol RNA** | | | **MWa, g/mol** | **mmol/g RNA** | **mmol/g DCW** |
| --- | --- | --- | --- | --- | --- | --- |
|  | **mRNA 0.05** | **rRNA 0.75** | **tRNA 0.20** |  |  |  |
|  |  |  |  |  |  |  |
| ATP | 0.1921 | 0.2256 | 0.1938 | 329.20 | 0.6749 | 0.0904 |
| GTP | 0.3083 | 0.2884 | 0.3063 | 345.20 | 0.9089 | 0.1218 |
| CTP | 0.3066 | 0.2718 | 0.3137 | 305.20 | 0.8746 | 0.1172 |
| UTP | 0.1930 | 0.2142 | 0.1862 | 306.20 | 0.6438 | 0.0863 |
| average |  |  |  | 306.2313 |  | 0.4157 |

Table 5 Phospholipids composition

| **Component** | **mol/mol phospholipids** | **after modified** | **MV, g/mol** | **g/g DCW** | **mmol/g DCW** |
| --- | --- | --- | --- | --- | --- |
| phosphatidylcholine (PC) | 0.3 | 0.317 | 275.304 | 0.0099 | 0.0360 |
| cardiolipin (CL) | 0.271 | 0.286 | 498.6310 | 0.0166 | 0.0333 |
| phosphatidylethanolamine (PE) | 0.25 | 0.264 | 270.2620 | 0.0078 | 0.0289 |
| phosphatidylglycerol (PG) | 0.125 | 0.132 | 900.6360 | 0.0040 | 0.0044 |

Table 6 Fatty acids composition

| **Component** | **mol/mol fatty acids** | **after modified** | **MV, g/mol** | **g/g DCW** | **mmol/g DCW** |
| --- | --- | --- | --- | --- | --- |
| Hexadecanoic acid | 0.260 | 0.286 | 256.42 | 0.0083 | 0.0324 |
| Octadecenoic acid | 0.600 | 0.659 | 281.00 | 0.0214 | 0.0762 |
| Octadecanoic acid | 0.050 | 0.055 | 284.48 | 0.0018 | 0.0063 |
| Glycerol | / | / | 92.09 | 0.0038 | 0.0413 |

Table 7 Peptidoglycan and Carbohydrates composition

| **Component** | **MV, g/mol** | **g/g DCW** | **mmol/g DCW** |
| --- | --- | --- | --- |
| NAG | 221.210 | 0.0146 | 0.0660 |
| NAM | 293.270 | 0.0146 | 0.0498 |
| Carbohydrates | 161.857 | 0.1752 | 1.0824 |

**References**

[1] Heefner D L, Claus G W. Lipid and fatty acid composition of Gluconobacter oxydans before and after intracytoplasmic membrane formation.[J]. Journal of Bacteriology, 1978, 134(1):38-47.

[2] Heefner D L, Claus G W. Change in quantity of lipids and cell size during intracytoplasmic membrane formation in Gluconobacter oxydans.[J]. Journal of Bacteriology, 1976, 125(3):1163-1171.

[3] Olijve W, Kok J J. An analysis of the growth of Gluconobacter oxydans in chemostat cultures[J]. Archives of Microbiology, 1979, 121(3):291-297.

[4] Wu X, Wang X, Lu W. Genome-scale reconstruction of a metabolic network for Gluconobacter oxydans 621H.[J]. Bio Systems, 2014, 117(2):10–14.
